# Characterization of the Mechanism of Inhibin α-Subunit Gene in Mouse Anterior Pituitary Cells by RNA Interference

**DOI:** 10.1371/journal.pone.0074596

**Published:** 2013-10-03

**Authors:** Li Han, Canjie Wu, Hasan Riaz, Liya Bai, Jianguo Chen, Yanhong Zhen, Aizhen Guo, Liguo Yang

**Affiliations:** 1 State Key Laboratory of Agricultural Microbiology, Laboratory of Animal Infectious Diseases, College of Animal Science and Veterinary Medicine, Huazhong Agricultural University, Wuhan, Hubei, China; 2 Key Lab of Agricultural Animal Genetics, Breeding and Reproduction of Ministry of Education. College of Animal Science and Veterinary Medicine, Huazhong Agricultural University, Wuhan, Hubei, China; The University of Queensland, Australia

## Abstract

Inhibin, a member of the transforming growth factor-β [TGF-β] superfamily, is a suppressor of follicle-stimulating hormone [FSH] release through pituitary–gonadal negative feedback loop to regulate follicular development. In this study, Inhibin α-subunit [*Inha*] gene was knocked down successfully in mice primary anterior pituitary cells at both transcriptional and translational levels by RNAi-Ready pSIREN-RetroQ-ZsGreen Vector mediated recombinant pshRNA vectors. The results indicated that inhibin silencing significantly promoted apoptosis by up-regulating Caspase-3, Bax and Bcl-2 genes without affecting p53 both at transcriptional and translational levels. Furthermore, it markedly impaired the progression of G1 phase of cell cycle and decreased the amount of cells in S phase [as detected by flow cytometry]. Inhibin silencing resulted in significant up-regulation of mRNA and protein expressions of Gondotropin releasing hormone receptors [GnRHR] and down-regulated mRNA levels of β-glycans with parellel change in the amount of its protein expression. Silencing of inhibin-*a* significantly increased [P<0.05] activin-β concentration without affecting FSH and LH levels in anterior pituitary cells. These findings revealed that up regulation of GnRH receptors by silencing inhibin *a*-subunit gene might increase the concentration of activin-β in the culture medium. Inhibin *a* silencing resulted in increased mRNA and protein expressions of inhibinβ which may demonstrate that both inhibin subunits co-participate in the regulation of reproductive events in anterior pituitary cells. This study concludes that inhibin is a broad regulatory marker in anterior pituitary cells by regulating apoptosis, cellular progression and simultaneously by vital fluctuations in the hormonal signaling.

## Introduction

Inhibin, a disulfide-linked dimer, plays an important role in the modulation of whole reproductive axis and encompasses all reproductive events [1]. In females, it is produced by ovarian follicles, and serves as a major antagonist in the control of FSH release. Inhibin molecule is composed of two subunits, α subunit and two β subunits independently encoded by separate genes [2]. The domination of inhibin α-subunit in controlling different reproductive functions has been previously described in many studies [3]. Previous work from our lab has described that inhibin DNA vaccine is promising for improving fertility in rats and goats [4]–[5].

Many previous studies have demonstrated the presence of inhibin α-subunit mRNA and protein expressions in the anterior pituitary cells [1], [6]–[7]. The proposed function of inhibin α-subunit is to primarily modulate FSH production through its endocrine feedback module [7]. Simialrly, its involvement in intra-ovarian paracrine system leads to the regulation of FSH and activin in pituitary gonadotrophs [8]. These gonadotrophs are dominated by inhibin co-receptor, betaglycan suggesting that inhibin might contribute its functions by altering activin, FSHβ and GnRH receptor expressions [9]–[10]. In addition to this, presence of beta glycan also implicates its autocrine exertion in pituitary cells, thereby, turns out to be a regulator of reproductive cycles [1]. This raises the question of whether production of inhibin in pituitary cells has functional association with local different steroids and their receptors [11]–[12].

Recently, RNA interference (RNAi) has proved its efficiency in modulation and alteration of a particular gene knock down model in many species [13]. In our previous work, we have successfully demonstrated the inhibin α-subunit silencing in mice Sertoli cells [14]. Not surprisingly, after silencing genetic modifications or alterations in the expression of inhibin α-subunit would result in varying degree of anomalies, due to its extensive association with local factors in anterior pituitary cells. Thus, there is a dire need to demonstrate its local regulation in pituitary cells particularly its local production with relation to FSH regulation.

Therefore, this study was designed to reveal the role of inhibin α subunit in cell cycle control and apoptosis of mice anterior pituitary cells. which was further verified by associated mRNA and proteins expressions. Furthermore, its involvement in regulation of different steroid hormones and its receptors in primary anterior pituitary cells were also addressed. The study would be better the understanding of inhibin α subunit local regulation within pituitary cells and overall, in reproductive events.

## Materials and Methods

### Experimental animals and antibodies

Immature female Kunming mice (SPFgrade, 30 days old) were purchased from Center of Laboratory Animals of Hubei Province, Wuhan, China and were housed in a single room under conditions of constant temperature (20–25°C) and humidity (60–75%), with a 12-h light/dark cycle. This study was approved by the Ethical Committee of the Hubei Research Center of Experimental Animals (Approval ID: SCXK (Hubei) 2008-0005). In this study, animals were treated in accordance with the NIH Guide for the Care and Use of Laboratory Animals. All the protocols had the approval of the Institutional Committee on Animal Care and Use.

Rabbit monoclonal antibodies including anti-inhibinα (EPY2782), anti-GnRHR (EPR5293, 1∶1000), anti-bax (1063-1, 1∶1000) and anti-bcl-2 (1017-1, 1∶1000) were purchased from Abcam, California, USA. All the other antibodies including anti-actin rabbit monoclonal antibody (SC-1616r, 1∶1000), anti-FSHβ goat polyclonal antibody (SC-7797, 1∶500), anti-β-glycan goat polyclonal antibody (sc-6199, 1∶1000), anti-LHβ mouse monoclonal antibody (SC-374017, 1∶500) and anti-p53 mouse monoclonal antibody (SC-55476, 1∶500) were purchased from the Santa Cruz Biotechnology, Inc. Texas, USA.

### Culture of the mouse anterior pituitary cells

Anterior pituitary glands were collected from 15 Kunming mice following anesthesia with sodium pentobarbital and exsanguination [15]. Briefly, under sterile conditions, pituitary glands were removed and the anterior lobe was immediately dissected from each pituitary gland. Fifteen-anterior pituitary glands were washed with the Dulbecco's Modified Eagle's Medium/Nutrient Mixture F12 (DMEM/F12)(Gibco,US) supplemented with 100 IU/mL penicillin, 100 µg/mL streptomycin, 2 mg/mL of BSA. Then, a pool of 15-anterior pituitaries was minced for suspension in the same medium followed by centrifugation for 10 min at 2000 rpm. After the removal of the upper supernatant, the lower sliced fragment was incubated at 37°C in the DMEM/F12 containing 0.25% trypsin-EDTA (Gibco, US) and 0.25% collagenase type II in a flask with constant stirring for 30 min. Then, the enzyme-digested pituitary suspension was centrifuged at 1500 rpm for 5 min, and the supernatant was discarded and the cell pellet was re-suspended with the medium. The cell suspension was then filtered through 75 µm nylon screens (200 meshes) to remove undigested tissue and cell aggregates and centrifuged at 1500 rpm for 10 min. The supernatant was discarded and the cell pellets were washed twice by the medium DMEM/F12 supplemented with antibiotics, then the cell pellets were diluted to 3×10^5^ live cells/mL with DMEM/F12 medium with 15% fetal calf serum (GIBCO, USA), 100 IU/mL penicillin, 100 µg/mL streptomycin. Finally, 2 mL of cells suspension was seeded in 6-well plates (day of seeding = day 0 of culture), all cells were cultured at 37°C in a humidified atmosphere containing 5% CO_2_.

### Immunohistochemistry staining (IHC Staining) for the inhibin α-subunit in the anterior pituitary gland

The expression of inhibin α-subunit in anterior pituitary cells was assessed in accordance with standard streptavidin-biotin-peroxidase complex procedures. Briefly, anterior pituitary gland was quickly removed, fixed in formalin solution, and was dehydrated in decreasing concentrations of ethanol. After clearing with xylene, paraffin embedded sections (6 µm thick) were dewaxed and were saturated with blocking solution for 30 min at room temperature (1% BSA, Wuhan Bio-Lengend Company). Following antigen unmasking, the sections were incubated with anti-inhibinα (1∶200, Rabbit monoclonal antibody, Epitomics, California, USA) at 4°C overnight. On the following day after being washed three times with PBS-T, the sections were then incubated with biotin-conjugated anti-rabbit secondary antibody and peroxidase-conjugated streptavidin (Kirkegaard& Perry Laboratories, USA). The negative control was obtained by replacing the primary antibody with phosphate buffer saline (PBS). Positive expression of inhibin α-subunit protein was defined as the presence of brown granules in the cytoplasm or stroma.

### Identification of mouse anterior pituitary cells by indirect Immunofluorescence (IF)

For IF, the mouse anterior pituitary cells were cultured in 35 mm Glass Bottom Cell Culture Dish with standard culture medium and fixed in 4% paraformaldehyde (Wuhan Boster Bio-Engineering Limited Company, China) for 30 min. After permeabilization with 0.04% Triton X-100 (Sigma, USA) for 10 min, cell were soaked in blocking solution (1% BSA) for 30 min at room temperature. Then, the cells were incubated with anti-inhibin α antibody (1∶200, inhibin alpha chain rabbit monoclonal antibody Epitomics,), diluted in PBS/5% bovine serum albumin (BSA), for 1 h at 37°C. Following three washings with PBS, cells were saturated with appropriate fluoresce in isothiocyanate (FITC)-conjugated anti-rabbit immunoglobulin (1/100, Boster, Beijing, China, diluted in PBS/5% BSA), for 30 min at room temperature in the dark. Controls were prepared omitting the primary antibody and replaced with PBS. Reactions were read by using a magnification of X315 and incident light at wavelengths of 450 to 490 nm for FITC fluorescence (green). Images were taken on an Axio Observer Z1 fluorescence microscope from Zeiss (Germany).

### Contruction and Transfection of Recombinant pSIREN-RetroQ-ZsGreen Vectors

Construction of inhibin plasmids used in this study was previously reported (14). Briefly, three siRNA target sites were selected from coding sequence of mouse inhibin alpha (Inha, BC056627) according to the siRNA program [16]–[17] at position 273, 772, and 1237 in the coding region. To obtain short hairpin RNA, a typical oligonucleotide that has 5 bases containing a restriction site at its 5′ end, 19 bases of sense strand, 7 to 9 bases of hairpin loop, 19 bases of antisense strand, 6 bases of terminator, and 6 bases corresponding to a unique HindIII restriction site (resulting in a total length of 65 bases) and 2 complementary oligonucleotides were synthesized. These were annealed and inserted into the BamHI and EcoRI sites of the RNAi-Ready pSIREN-RetroQ-ZsGreen Vector (BD Biosciences, Clontech, Mountain View, CA). The recombinant plasmids were designated as pshRNA-1, pshRNA-2, and pshRNA-3, respectively. A plasmid (pshRNA-negative) encoding a hairpin siRNA comprising a nonsense sequence that has not been found in the mouse or human genomes was used as the negative control.

Plasmids of pshRNA-1, 2, 3 and pshRNA-negative were recycled using EndoFree Plasmid Kit (Tiangen, Beijing, China), and confirmed by respective sequencing. These vectors independently expressed a Zoanthus sp. green fluorescent protein [18], which had been engineered for brighter fluorescence (excitation maximum = 496 nm; emission maximum = 506 nm). As a result, the transfected cells emitted green fluorescent protein.

One day before transfection, 0.5–2×10^5^ cells were seeded to 70–80% confluence in 12-well plate. Four groups of anterior pituitary cells were prepared in total to transfect pshRNA-1, 2, 3, and pshRNA-negative, respectively. The transfection procedure was performed using Lipofectamine™ LTX with Plus™ Reagent (Invitrogen) according to manufacturer's instructions. After 8 h, transfection medium was changed into fresh growth medium without antibiotics. Cells were collected for RNA and protein extraction in respective time intervals, and the culture medium was collected and preserved for detection of hormones.

### RNA extraction and Real-Time PCR

Anterior pituitary cells were transfected with the vectors, pshRNA-2, pshRNA-negative, and PBS respectively. After 48 h, cells were washed in PBS and total cellular RNA was extracted using RNAprep pure cell Kit (Tiangen, Beijing China). For the removal of residual genomic DNA, these samples were treated with DNaseI. The first-strand cDNA was synthesized using first strand cDNA synthesis kit (code NO.FSK-100; Toyobo Co. Japan) and quantitative real-time PCR was carried out using SYBRGreen (SYBR Green Real time PCR Master Mix QPK-201; Toyobo Co Japan.). Specific PCR settings were used in a Bio-Rad iQ5 Real Time PCR system. To verify PCR product purity, samples were subjected through melting curve analyses after real-time PCR reactions. Primer pairs were summarized in Table 1, The threshold cycle (CT) numbers were calculated for the amplified cDNA for each investigated mRNA and for the housekeeping gene (β-actin) in each sample. The relative mRNA expression levels were estimated using the formula: 2−ΔΔCT (Livak and Schmittgen, 2001).

**Table 1:** Sequences of primer pairs for quantitative real-time PCR.

Gene name	Forward Primer sequences [5′–3′]	Reverse Primer sequences [5′–3′]	Length
Inha	GAACCAGAGGAGGAAGATGTC	CCAGATGATAGCACCAGAAGA	260
Actb	CTGAGAGGGAAATCGTGCGT	CCACAGGATTCCATACCCAAGA	208
Bcl2	CGAGAAGAAGAGAGAATCACAGG	AATCCGTAGGAATCCCAACC	133
Bax	AGGATGCGTCCACCAAGAA	CAAAGTAGAAGAGGGCAACCAC	195
Casp3	TGACTGGAAAGCCGAAACTC	GCAAGCCATCTCCTCATCAG	101
p53	CGACCTATCCTTACCATCATC	GGGTGAAATACTCTCCATCAAG	244
Inhbb	TCAGCTTTGCAGAGACAGAT	TCTTGGAAGTACACCTTGACC	184
FSHb	TTGACCAACATCACCATCTC	ACTGGATATGTGTAGAGGGAGTC	221
LHb	TACTGTCCTAGCATGGTCCG	AGGGCTACAGGAAAGGAGAC	149
GnRHR	TCAAGACCCACGCAAACTAC	GATTCACTGGCTCTGACACC	188
Tgfbr3	CTGAATGGCTGTGGTACTAGAC	CGACTCCAAATCTTCGTAGC	132

### Western blotting Analysis

The anterior pituitary cells transfected with the recombinant RNAi vectors were scraped off 48 h after transfection, washed in cold PBS and lyzed in RIPA buffer (Santa Cruz, USA) containing protease inhibitor cocktail (Santa Cruz, USA). After 1 h incubation at 4°C, cells were centrifuged at 12000 g for 10 minutes for the removal of cellular debris, respectively. Total protein concentrations were ascertained by BCA-assay (Pierce, Rockford, USA), and 50 µg of total protein was submitted to gel electrophoresis. Proteins were separated on a 12% polyacrylamide gel before transferring them to PVDF membranes (Millipore, Bedford, MA). After blocking in PBS supplemented with 5% skim milk (Sigma-Aldrich) and 0.05% Tween 20 (Sigma-Aldrich), membranes were incubated overnight at 4°C with anti-inhibin α rabbit monoclonal antibody (1∶1000). After incubation with the primary antibody, membranes were washed three times with PBS containing 0.1% Tween 20, incubated for 1 h with 3000-fold diluted HRP labeled goatanti-rabbit secondary antibodies (Kirkegaard& Perry Laboratories) at room temperature and washed three times with pre-warmed PBS containing 0.1% Tween 20. After washing, blots were developed using the ECL Western Blotting detection system (Amersham Biosciences, Piscataway, NJ), and then exposed to X-ray film for visualization of the protein bands. PVDF blots were then stripped of bound antibodies and treated with mouse β-actin antibody (1∶1000 dilutions; Santa Cruz Biotechnology, Santa Cruz, CA, USA) for normalization. The band intensities were measured with AlphaEaseFC software (AlphaInnotech, USA)

### Flow cytometry and cell cycle analysis

Anterior pituitary cells transfected with different RNAi vectors were harvested at respective time intervals, washed with PBS, fixed in ice-cold 75% ethanol overnight at 4°C, washed again in PBS and stained using propidium iodide/RNase A solution at 37°C in the dark chamber for 30 min. Flow cytometric analyses were conducted using a BD FACSCalibur (Becton, Dickinson and Company, USA) and ModFit LT for Mac V3.0 software. For each determination, a minimum of 20,000 cells was analyzed. All experiments were repeated independently five times.

### Detection the cell apoptosis by Flow cytometry

After transfection anterior pituitary cells were cultured in 12-well plates for 48 h, washed with PBS and then harvested by digestion with trypsin at 37°C for 5 min. Cell apoptosis was probed with Annexin V-APC/propidium iodide (PI) and detected by flow cytometry (BDFACSCalibur, USA) according to the manufacturer's instruction (Apoptosis Detection Kit, KeyGEN, Nanjing). Viable cells were negative for AnnexinV-APC and propidium iodide stain; early apoptotic cells were positive for Annexin V- APC stain and negative for propidium iodide stain; late apoptotic cells were double stained by Annexin V-APC and propidium iodide. Experiments were repeated five times independently.

### Effects of INHA regulation on expression of p53, Bcl2, Bax, Casp3, INHβB, FSHβB, LHβB, GnRHR, β-glycan

To determine whether *INHA* had an association with members of TGFβ superfamily, relative mRNA expressions of p53, Bcl2, Bax, Casp3 (apoptotic related genes), INHβB, FSHβB, LHβB, GnRHR (Steroid hormone receptors) and β-glycan (growth factor) were quantified by real-time PCR among pshRNA-2, pshRNA-negative and PBS groups, respectively.

### Reproductive hormones analyzed by RIA (Radioimmunoassay)

After transfection with pshRNAi-2, pshRNA-negative and PBS, the culture medium was collected every 6 h over 48 h to measure concentrations of FSH, LH, INHB and ACVB (Activin B) using the mouse radioimmunoassay kits and ELISA kits. The hormonal concentrations were measured by double-antibody radiommunoassay (RIA) system using Iodine [^125^I]-labeled hormones radioimmunoassay kit according to its instructions (Beijing North Institute of Biological Technology, BNIBT, China).

The sensitivity of FSH and LH RIA was 0.27 mIU/mL and 0.4 mIU/mL respectively. Similarly, the sensitivity of inhibin-B and Activin-B ELISA kit (Wuhan ColorfulGene biological technology Co., Ltd, China) was 2 ng/L–40 ng/L and 3 pg/ml–180 pg/ml respectively.

### Statistical Analysis

Changes in the concentrations of inhibin, FSH, LH were subjected to one-way ANOVA. Level of significance was determined by Duncan's multiple range tests. All data were analyzed using the General Linear Models Procedure of Statistical Analysis Systems (SAS Inc., Cary, NC, USA). A value of P<0.05 was considered to be significant. All data is represented as mean ± SEM of repeated experiments (n = 3).

## Results

### Expression of the inhibin α-subunit on primary anterior pituitary cells

**Figure 1:**
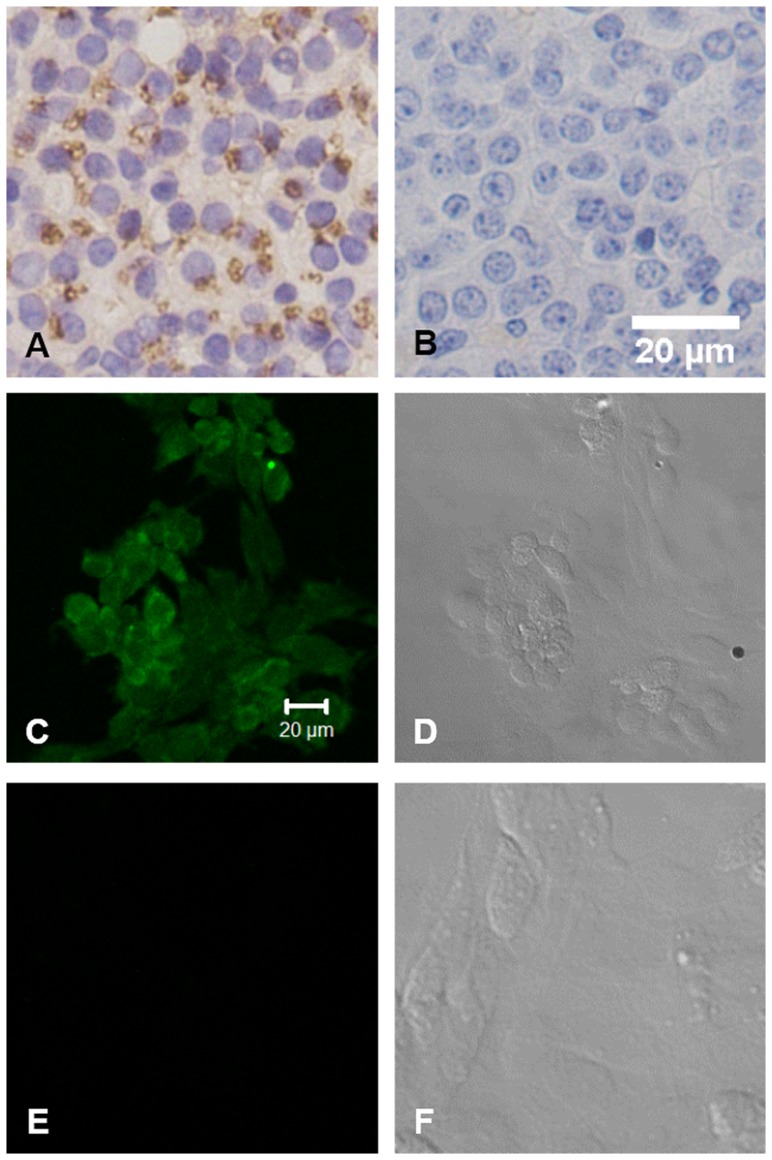
Detection of inhibin α-subunit expression in the mouse anterior pituitary. A: Inhibin α-subunit expression in the mouse anterior pituitary gland using immunohistochemical methods. Immunohistochemical analysis of inhibin α subunit expression in the anterior pituitary gland by inhibin monoclonal antibody [A1] and PBS [A2], magnification is ×40. B: Inhibin α subunit expression in the cultured mouse anterior pituitary cells using indirect immunofluorescence. Indirect immunofluorescence analysis of inhibin α subunit expression in the anterior pituitary cells by inhibin monoclonal antibody [B1] and PBS [B2], magnification is ×40.

The presence of inhibin *α*-subunit protein expression was first confirmed by immunohistochemical analysis. The result showed the appearance of brownish granules scattered within pituitary stroma (Fig. 1). To further reveal the cell-level distribution of inhibin ***α***-subunit expression, cells were cultured and subjected to immunoflourescence assay. The cells were incubated with the primary protein anti-inhibin ***α***-subunit mAb and then with a secondary FITC-conjugated antibody. Fluorescence was detected either by microscopy. These results indicated the presence of inhibin ***α***-subunit at cellular level in mice anterior pituitary gland.

### The pshRNA-2 induced the best silencing effect in the APCs

**Figure 2:**
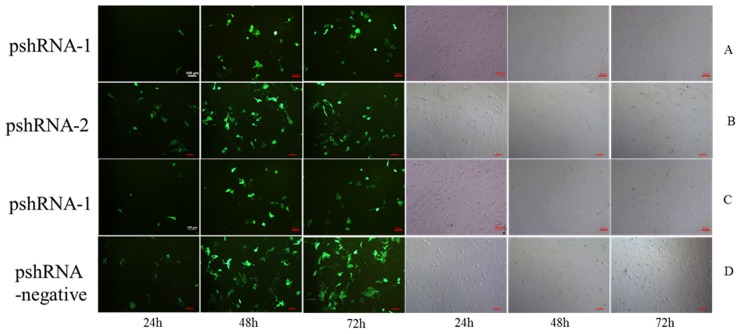
Inhα RNAi recombinant plasmids expression in the anterior pituitary cells. Three Inhα RNAi recombinant plasmids were transfected in anterior pituitary cells named as pshRNA-1, pshRNA-2, pshRNA-3 and pshRNA-negative, respectively for 24 h, 48 h or 72 h, the best efficient of which was selected for further investigation.

**Figure 3:**
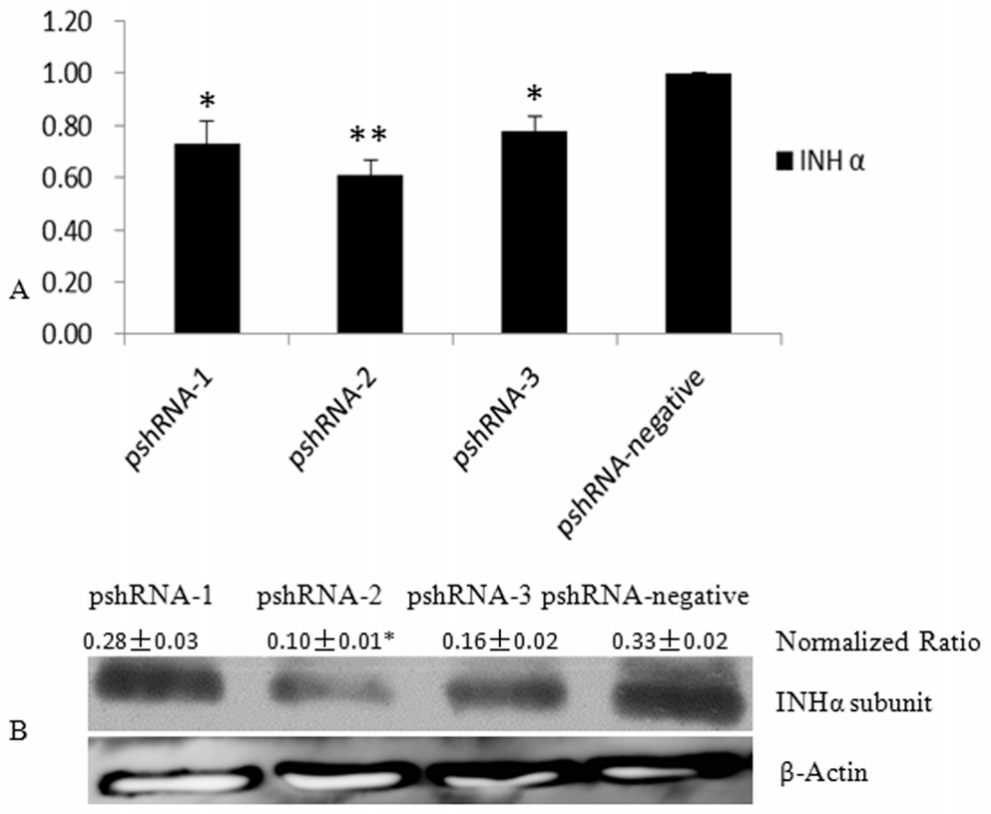
Detection of INH α subunit in the anterior pituitary cells after 48 h transfected with pshRNA-1, pshRNA-2, pshRNA-3 and pshRNA-negative, respectively. A: Transcription levels of INH α gene in the anterior pituitary cells transfected with pshRNA-1, pshRNA-2, pshRNA-3 and pshRNA-negative respectively. The statistical differences were tested using one-way ANOVA. Asterisk [*] means P<0.05, two asterisks means P<0.01. **B:** INHα subunit expression were detected by western blotting in the anterior pituitary cells after 48 h transfected with pshRNA-1, pshRNA-2, pshRNA-3 and pshRNA-negative respectively.

For further progression of the experiment, we intended to select the most efficient plasmid for anterior pituitary cells. We constructed three recombinant plasmids against inhibin α-subunit and tranfected them in the primary anterior pituitary cells. After 48 h, we detected their down-regulation efficiency by extent of their transfection ability (as measured by green fluorescence in cultured cells), level of mRNA (by q-PCR), and expressions of protein (by western blotting). The results showed that all three plasmids were able to down-regulate inhibin α-subunit expression, while all three parameters (presence of green fluorescence in cells as shown in Fig. 2, transcriptional and translational levels as shown in Fig. 3) in agreement depicted the higher down regulation of mRNA and protein expression by pshRNA-2 (60% down regulation) as compared to other plasmids thus ascertained the inhibition of expression at protein level.

### Silencing INHA changed Growth and Proliferation of the mouse primary anterior pituitary cells

**Figure 4:**
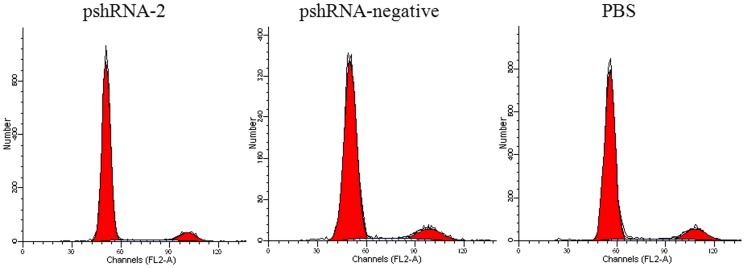
Cell cycle analysis of mouse pituitary cells by flow cytometry. The results of cell cycle examination demonstrated that the number of cells in S phase was significantly decreased in pShRNA2 group [p<0.05, compared with pShRNA-negative and PBS groups], indicating S phase arrest.

To check whether inhibin α-subunit is involved in the regulation of cell cycle progression in anterior pituitary cells, we saturated anterior pituitary cells with propidium iodide (PI) that usually stained the nuclear contents of a cell. Then, the cells were subjected to fluorescent activated cell sorter (FACS). The results showed that inhibin α-subunit significantly arrested (P<0.05) the G1 phase of cell cycle when compared with pshRNA-negative and PBS (Table 2; Fig. 4). This led to significant decline in the S phase in pshRNA-2 group compared with control groups. These results showed that inhibin α-subunit had a crucial role in the growth progression of anterior pituitary cells.

**Table 2:** Analysis of Cell Cycle by Flow Cytometry in the anterior pituitary cells 48-2, pshRNA-negative and PBS [Mean ± SEM, n = 3].

Groups cell cycels	G0/G1 [%]	S [%]	G2/M [%]
pshRNA-2	87.08±0.38*	8.54±0.06*	4.39±0.32
pshRNA-negative	83.77±0.08	11.50±0.36	4.74±0.28
PBS	82.95±0.16	11.62±0.15	5.44±0.31

All results were evaluated by One-way ANOVA.

[*] indicates level of significance in columns [P<0.05].

### Silencing INHA induced mouse pituitary cells apoptosis

To elucidate the role of inhibin α-subunit in regulation of apoptosis in anterior pituitary cells, we detected the exposure of phosphatidylserine on the cell surface with AnnexinV-APC/PI double staining following transfection with pshRNA-2, pshRNA-negative or by simply adding PBS in cultured cells. The results depicted a significant increase in the apoptotic cells in pshRNA-2 transfected group when compared with control groups (Table 3). These results explained that inhibin α-subunit increase the apoptotic cells thus, indicated as a strong apoptotic inducer in mice anterior pituitary cells.

**Table 3:** Measurement of mouse pituitary cell apoptosis.

Groups	Alive cells	Necrotic cells	Apoptotic cells
pshRNA-2	81.62±1.47**	7.39±0.74	8.77±0.51*
pshRNA-negative	87.76±1.16	5.08±0.56	3.58±0.96
PBS	84.98±1.23	6.40±0.65	5.80±2.09

There were significantly less apoptotic cells and more vital cells in the pShRNA-2 group compared to those of pShRNA-negative and PBS group respectively. [Means±SE, n = 3].

All results were evaluated by One-way ANOVA,

[*] P<0.05

[**] P<0.01 indicates level of significance in columns.

### Silencing INHA regulated the expression of p53, Bcl2, Bax, Casp3, INHβB,FSHβB, LHβB, GnRHR, β-glycan

**Figure 5:**
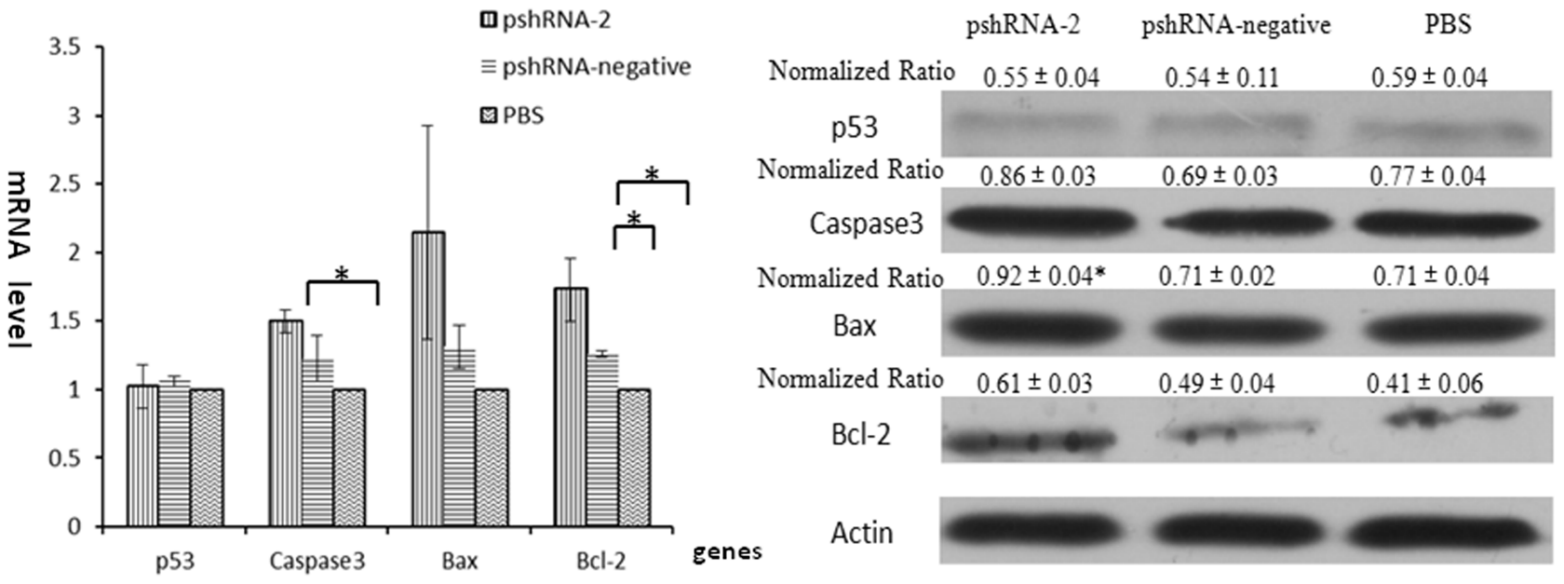
Levels of mRNA and protein expressions of apoptosis related genes were detected by q-PCR and western blotting. A: The mRNA levels of apoptosis related genes were examined by q-PCR. The results demonstrated that Caspase-3 and Bcl-2 was significantly decreased in pshRNA2 group 48 h post-transfection [p<0.05, compared with pshRNA-negative and PBS groups] but the levels of Bax and p53 gene was unlatered [p>0.05]. B: The protein levels of apoptosis related genes were detected by western blotting. The results showed that Caspase-3, Bax and Bcl-2 genes was up-regulated by inhibin gene silencing. But p53 gene was not affected by inhibin silencing.

To further validate the role of inhibin α-subunit on apoptotic factors, we quantified the mRNA and protein expressions of p53, Caspase-3, Bax andBcl-2. The results showed that inhibin α-subunit significantly up-regulated mRNA and protein expressions of caspase-3 (P<0.05) and bcl-2 (P<0.05). However, no significant difference was found in p53 and bax (P>0.05) gene expressions (Figure 5). These finding suggested that inhibin α-subunit prevented apoptosis through casp3-dependent pathways in mice anterior pituitary cells.

**Figure 6:**
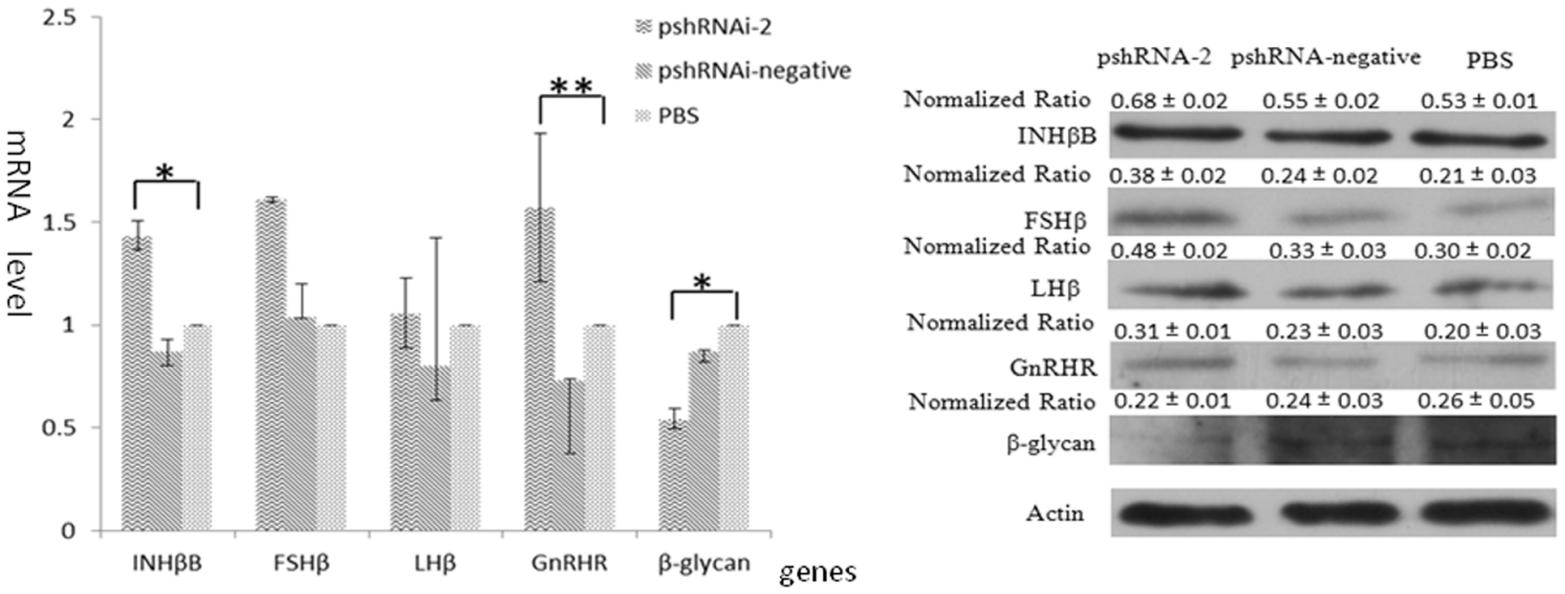
Effects of inhibin silencing on INHβB, FSHβB, LHβB, GnRHR and β-glycan. The levels of INHβB, FSHβB, LHβB, GnRHR and β-glycan were quantified by real-time PCR and western-blotting among pshRNAi-2, pshRNAi-negative and PBS groups, respectively. **A:** The mRNA levels of INHβB, FSHβB, LHβB, GnRHR and β-glycan were examined by q-PCR. Compared with pshRNA-negative and PBS groups, the mRNA levels of INHβB, FSHβB, LHβB and GnRHR were up-regulated [P>0.05] in APCs transfected with pshRNAi-2. However,mRNA levels of INHβB and GnRHR were significantly up-regulated, whereas mRNA level of β-glycan was significantly down-regulated. B: The proteins levels of FSH, LH, ACVB, INHB, GnRHR and β-glycan were detected by western blotting. The results showed that FSH, LH, ACVB, INHB, GnRHR proteins were up-regulated by inhibin gene silencing. But the protein of β-glycan was down-regulated by inhibin silencing.

We also determined the potential relationship of inhibin α-subunit in the regulation of steroid hormone receptors and TGFβ super family through their mRNA and protein expressions. Real-time PCR results depicted an increase in mRNA levels of all steroid receptors, and, inhibin α-subunit silencing resulted in significant up-regulation of mRNA levels of INHβB and GnRHR genes in APCs when compared with controls. Moreover, mRNA level of β-glycan showed significant down regulation after inhibin α-subunit silencing. As shown in Figure 6, similar results were obtained when we determined the protein expressions of these receptors and growth factors.

### Silencing INHA altered the hormones secretions of FSH, LH,inhibin B and activin B in APCs

**Figure 7:**
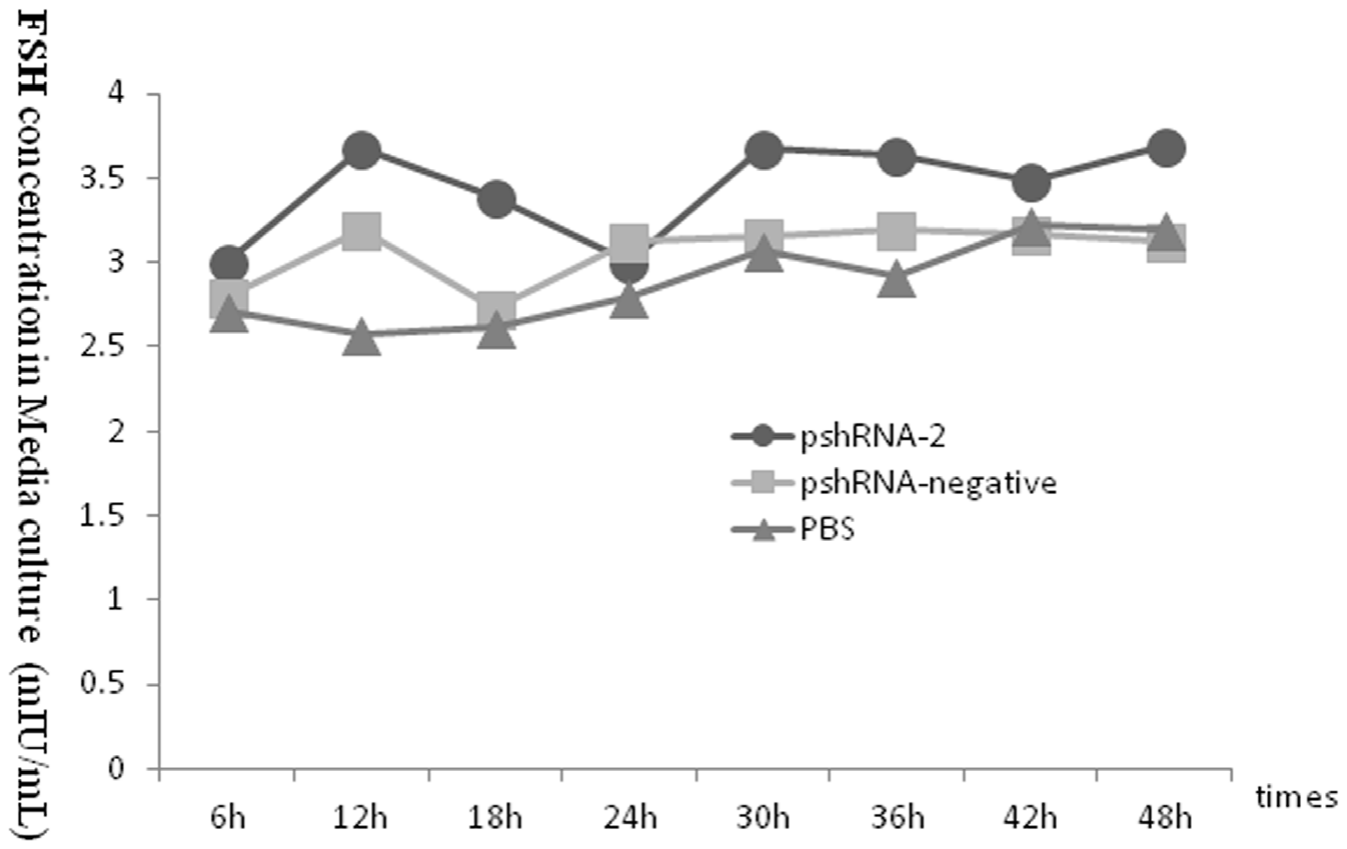
Concentration of FSH in the culture medium released by the mouse anterior pituitary cells at the different times after transfection. The results indicated that the FSH concentration 48-transfection was higher in pshRNA-2 group than those of pshRNA-negative and PBS groups respectively.

**Figure 8:**
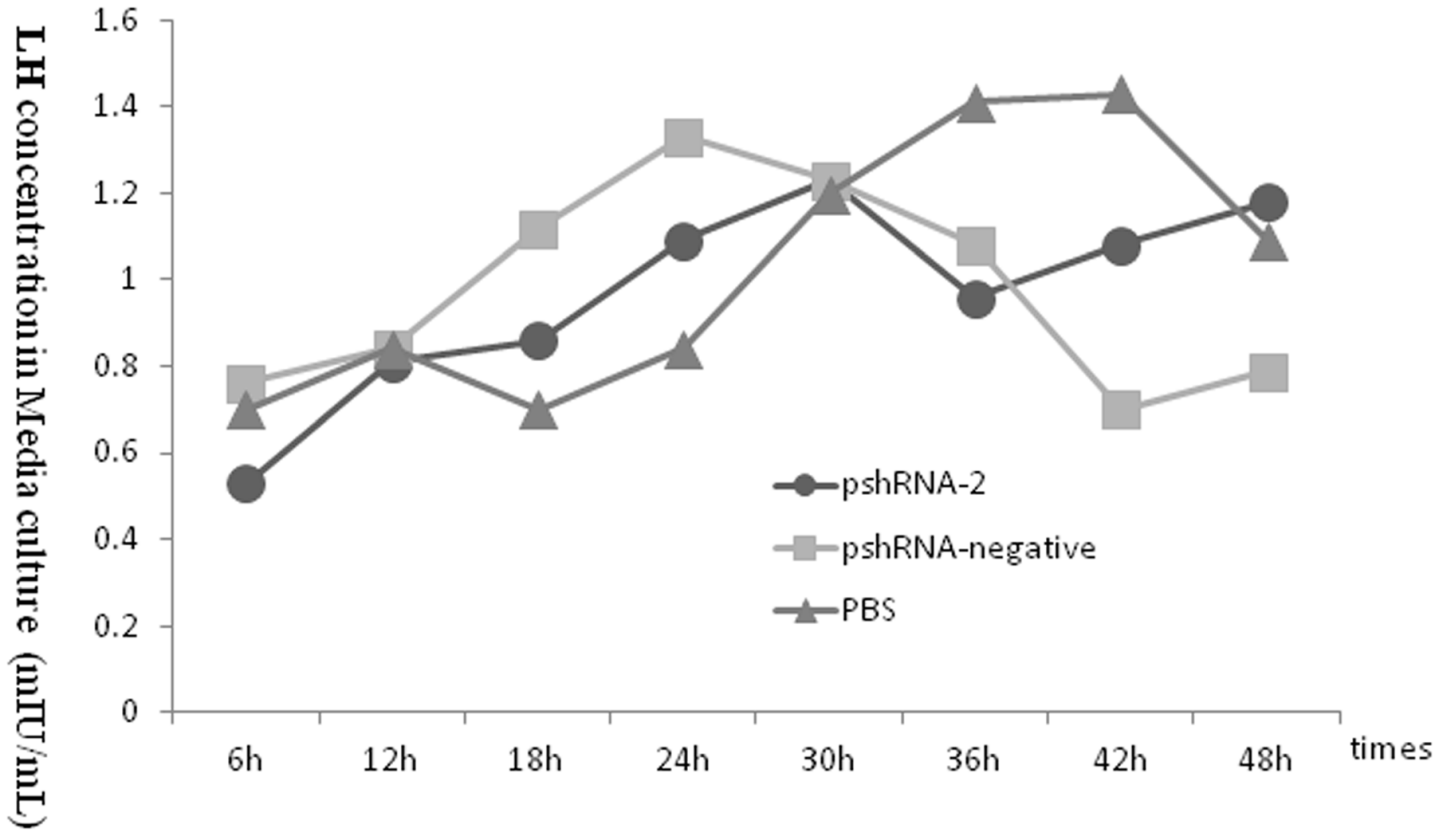
Concentration of LH in the culture medium released by the mouse anterior pituitary cells at the different times after transfection. The results indicated that the LH concentrations in the group were not affected by inhibin gene.

To assess the effect of inhibin α-subunit silencing on hormonal changes, we measured the concentrations of FSH, LH every 6 h up to 48 h. Furthermore, concentrations of INHB (inhibin B) and activin B (ACVB) were also measured 24 h and 48 h post-transfection. The results demonstrated that the release of FSH and LH in APCs transfected with pshRNA-2 was higher (P>0.05) than pshRNA-negative and PBS groups among different time intervals (as shown in Fig. 7 and 8).

**Figure 9:**
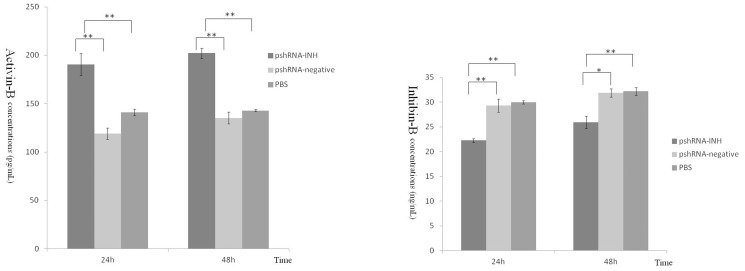
Concentration of INHB and ACVB in the culture medium released by the mouse anterior pituitary cells at the different times after transfection. A: Detection of the INHB concentration was done by RIA kit. Compared with pshRNAi-negative and PBS groups, the INHB concentrations was decreased significantly at 24 and 48 h after transfection with pshRNAi-2. B: Similarly, compared with pshRNAi-negative and PBS groups, the ACVB concentrations was increased significantly at 24 and 48 hours after transfection with pshRNAi-2.

Compared with pshRNAi-negative and PBS groups, the INHB (inhibin B) concentration was decreased significantly in APCs transfected with pshRNAi-2, at 24 h and 48 h. On the other hand, the ACVB concentration was increased significantly (P<0.05) in APCs at 24 and 48 h post transfection (as shown in Fig. 9). These results showed that decrease in INHB and and increase in ACVB concentrations can be associated with increase in FSH concentration in APCs transfected with pshRNAi-2 vector.

## Discussion

We report here the successful in-vitro transfection of inhibin α-subunit in mice anterior pituitary cells. After constructing three RNAi vectors, we determined their stable expression and transfection efficiency in these cells which resulted in the down regulation of mRNA and protein levels of inhibin α-subunit. Among these vectors, pshRNAi-2 performed efficiently in silencing the inhibin α-subunit mRNA and protein expressions, which helped us to further evaluate the mechanisms of anterior pituitary cells regulation, development and hormonal alterations.

The versatile effect of inhibin gene provoked us to check its role in the regulation of apoptosis and cell cycle regulations of anterior pituitary cells. Recently, inhibin α-subunit has been implicated in maintainace of cellular development and apoptosis in different cellular models [19]–[20]. Our results demonstrated that inhibin α-subunit silencing significantly promoted apoptosis in anterior pituitary cells and thus had important role in the suppression of programmed cell death in these cells. To further verify the apoptotic factors, we quantified mRNA and protein expression of apoptotic inducers (casp3 and p53), pre-apoptotic regulator (bax) and anti-apoptotic factor (bcl-2) in these cells. We found an increase in the expression of casp3, bax and bcl-2 both at transcriptional and translational levels without any significant alteration in p53 expression. This indicated that inhibin α-subunit silencing led to an imbalance in the bcl-2/bax family and promoted mitochondrial-mediated apoptosis [19]. Previous studies reported casp3-dependent repression of apoptosis in ovarian granulosa cells when treated with inhibin analogue [21]. Caspase-3 is an important effector molecule in promoting apoptosis in all types of cells. Its activation leads to initiation of cascade of caspases, responsible for execution of cells [14]. Another important regulator is p53, which regulates apoptosis and cell cycle suppression. In this study we did not detect significant alteration in mRNA and protein level of p53 gene. This inability of p53 in promoting the casp3-dependent apoptosis has been previously described in nervous system [22]. Similarly, silencing of inhibin α-subunit led to a significant arrest in G1 cell cycle phase which further diminished the cells in S/G2 phase. For normal cellular progression, p53 is vital growth stopper, which activates p21 and stops cellular progression at G1 phase. However, recently, p53-independent G1 cell cycle arrest was observed in adenocarcinoma cells which revealed the importance of c-Myc and p21 in the regulation of cell cycle progression [23]. This indicated that inhibin had very complex organization in controlling the cellular progression and apoptosis in mice anterior pituitary cells. Further studies are needed to elucidate the possible mechanisms behind these factorial alterations in these cells.

We quantified the relative mRNA and protein expression of inhβB, FSHβ, LHβ, GnRH receptors and beta glycan. In addition, we measured in-vitro hormonal secretions of FSH, LH and Activin-β following inhibin α-subunit silencing. The results showed a significant up regulation of mRNA expressions of GnRH receptors, and downregulation of β glycans. On the other hand, inhibin α-subunit silencing did not improve mRNA and protein levels of FSH and LH as well as their hormonal secretions. But, overall trend was towards increasing concentration of these hormones. Previous studies demonstrated the priming of betaglycans in the modulation of action of inhibins. Betaglycan knock out rats failed to attenuate the activin-stimulated FSH secretions, FSHβ expression and GnRH receptors [24]–[26]. In this study, it was evident that silencing of inhibin α-subunit disrupted the inhibin-beta glycan signaling pathway and therefore increased the expression of GnRH receptors and this resulted in upregulation of mRNA and protein expressions of FSHβ and hormone secretions in these cells. The activation of GnRH receptors has been proposed due to increase in bio-GnRH binding capacity [27]. These findings are in accordance with many previous findings that suggest inhibin regulation is important in steroid hormone regulation.

On the other hand, release of FSH can be affected by a number of other factors including gonadotophin inhibitory hormone (GnIH). In vitro treatment of ovine pituitary cell culture showed that GIRH can reduce the FSH response after GnRH activation [28]. Similarly, treatment with RFamide related peptide-3 (RFRP-3) reversed the GnRH-induced FSH and LH secretions in ovine pituatory gonadotrophs [29]. In another study, short-term treatment of GnIH (120 min) depressed FSH and LH release as well as down-regulated mRNA levels of FSH α and β-subunit mRNAs, without affecting mRNA level of LHβ [30]. This might be the possible reason for non-significant improvement in FSH secretions in this study.

Activin is documented as potent activator of FSHβ in synergy with pulsate release of GnRH under activin signaling pathway in pituitary cells which activate FSHβ and GnRH receptor promoters [31]–[33]. Furthermore, this exclusive mediation of activin-β is documented as autocrine/paracrine and therefore influences in the regulation of FSH secretions [34]. However inhibin has no effect on mRNA or protein expressions of LH and its hormonal secretions. Earlier studies addressed GnRH-mediated LH suppression in rats [35]. But, treatment with inhibin antagonist has no effect on LH releasing percentage area of pituitary cells [36].

These findings also verified the tendency of auto regulation within inhibin gene subtypes. In this study, silencing of inhibin α-subunit increased transcriptional and translational expression of inhbin β-subunit, which served as new evidence in the regulation of functions of anterior pituitary cells. This correlation has been previous described in mice adrenal glands and Sertoli cells [14], [37]. Further investigations are required to modulate this auto-regulation and to elucidate the inhibin β-subunit regulation in these cells. It is already reported that pituitary cells exhibited preferential sensitization to both inhibin subtypes [38].

In conclusion, our results disclosed that inhibin α-subunit was important in regulation of apoptosis and cell cycle progression in anterior pituitary cells. This was further confirmed by the associative apoptotic and cell cycle factors. Furthermore, inhibin α-subunit has a role in the regulation of steroid hormone biosynthesis and their receptors. Silencing of inhibin α-subunit interruptted its co-receptor β-glycan and increased GnRH receptor which subsequently increased mRNA and protein expressions of activinβ and its secretions, but had no significantly effect on the mRNA and protein expressions of FSH and LH and their hormonal secretions. Its auto-regulatory mechanism with its β-subtypes needs further investigations.
